# Comparing habitual and i. Scription refractions

**DOI:** 10.1186/s12886-019-1053-x

**Published:** 2019-02-12

**Authors:** Nicole M. Putnam, Balamurali Vasudevan, Andre Juarez, Cam Tu Le, Kristine Sam, Pablo de Gracia, Allissun Hoppert

**Affiliations:** Midwestern University, College of Optometry, 19555 N 59th Ave, Glendale, Arizona 85308 USA

**Keywords:** I.Scription, Night myopia, I.Profiler^plus^, Optical aberrations, Pseudomyopia

## Abstract

**Background:**

Many patients voice concerns regarding poor night vision, even when they see 20/20 or better in the exam room. During mesopic and scotopic conditions the pupil size increases, increasing the effects on visual performance of uncorrected (residual) refractive errors. The i.Scription refraction method claims to optimize traditional refractions for mesopic and scotopic conditions, by using the information that the Zeiss i.Profiler^plus^ gathers of ocular aberrations (low and high order). The aim of this study was to investigate any differences between habitual and i.Scription refractions and their relationship to night vision complaints.

**Methods:**

Habitual, subjective, and i.Scription refractions were obtained from both eyes of eighteen subjects. Low and high order aberrations of the subjects were recorded with the Zeiss i.Profiler^plus^. The root mean square (RMS) metric was calculated for small (3 mm) and maximum pupil sizes. Subjects rated their difficulty with driving at night on a scale of 1–10.

**Results:**

There was a statistically significant difference between the habitual and i.Scription refractions on both the sphere and cylinder values [(t = 3.12, *p* < 0.01), (t = 5.39, p < 0.01)]. The same was found when comparing the subjective and i.Scription refractions [(t = 2.31, *p* = 0.03), (t = 2.54, *p* = 0.02)]. There were no significant differences found when comparing the sphere and cylinder values between the habitual and subjective refractions or on any combination of spherical equivalent refraction. The maximum pupil size of the subject population on this study, measured with the i.Profiler^plus^, was 4.8 ± 1.04 mm. Ten out of the eighteen subjects had discomfort at night with an average magnitude of 4 ± 2.7. Ratings of difficulty with night vision correlated with the change in spherical equivalent correction between the habitual and i.Scription refractions (*p* = 0.01). A sub-analysis of myopic subjects (*n* = 15) showed an increase in the significance of this relationship (*p* = 0.002).

**Conclusions:**

The i.Scription method improves night vision by correcting the sphere and cylinder more precisely. There was a correlation between the amount of change in the cylinder value between habitual and i.Scription prescriptions and the magnitude of the reported visual discomfort at night.

**Electronic supplementary material:**

The online version of this article (10.1186/s12886-019-1053-x) contains supplementary material, which is available to authorized users.

## Background

It is not uncommon for patients to complain of poor night vision during a routine eye exam [1]. This is true even when a patient is able to see 20/20 or better in the exam room, especially with respect to night driving [2, 3]. The literature [3, 4] has shown that poor night vision is often due to a phenomenon called *night myopia*, where a person’s vision appears to become more nearsighted in low lighting conditions. Many factors may contribute to poor night vision in an otherwise healthy patient including uncorrected or under-corrected refractive error, increased accommodation, and an increase in ocular aberrations [4, 5].

Many factors influence night vision including but not limited to accommodation, aberrations, and pupil size. Lopez-Gil [6] reported that the accommodating eye to be significantly more myopic in a condition with a point source on a dark background when compared to the conventional dark letters on a bright background. An increase in optical aberrations resulting from the natural dilation of the pupil is another possible cause of night myopic shift [7–10]. The dilated pupil also lets in more light and a confounding factor could be that the resulting increased retinal illuminance could result in less visual discomfort from stimuli present during night driving [11, 12]. Interestingly, Faria-Ribeiro et al. [13] using a through focus Visual Strehl of the Modulation Transfer Function [VSMTF] criteria, reported that the foveal refractive error does not change much with an increase in pupil diameter, i.e., the increase in positive spherical aberration will lead to a degradation of lower spatial frequencies, that becomes more significant under low illumination levels. More recently, Marin-Franch et al. [14] investigated the effect of spherical aberration on tasks that are typically viewed with nighttime light levels and reported that small point sources will generate myopic shifts in subjective refractions, but only in the presence of positive spherical aberrations. In addition, an opposite effect is seen with negative spherical aberrations.

One of the only instrument of its type currently on the market, the i.Profiler^plus^ consists of an autorefractor and an aberrometer [15]. Patient’s subjective refraction measurements are obtained, and used in the accompanying software program. i.Profiler^plus^ will compute an i.Scription, a version of the manifest refraction accurate to 0.01D that has been minimally modified to account for the interaction of high-order aberrations with low-order aberrations. While high-order aberrations can’t be corrected directly, the spectacle prescription can be modified to account for the interactions of low-order with high-order aberrations: an i.Scription is generated from information gathered by the i.Profiler^plus^ by Zeiss. According to Zeiss, the purpose for the i.Scription is to provide improved color and contrast as well as better nighttime driving vision for the patient [16]. Another notable instrument approaches the problem from a different angle, the Vmax Vision PSF Refractor (Vmax Vision, Orlando, FL). The PSF Refractor incorporates the subjective refraction into the instrument. Patients view a PSF target rather than a conventional eye chart and the resulting refractions are accurate to 0.05D [17].

Thus, the aim of this study was to investigate the difference in the refractions generated by i.Scription computed from a manifest subjective refraction (results of a subjective refraction) and the habitual refraction (self-reported by the subjects). The i.Profiler^plus^ aberration values, pupil size measurements and the patients self-rated night vision complaints were also considered in relation to this refractive data.

## Methods

Eighteen subjects completed this prospective study from August 2014 to April 2015. Each subject was scheduled for a visit of no more than one hour. All the subjects participated on a voluntary basis. Subjects were recruited via a campus wide e-mail to all students, faculty and staff. Clinic patients at the Midwestern University Eye Institute were also be invited to join the study if they met the inclusion criteria. Subjects were of either sex and of any ethnic group. Inclusion criteria included being between the ages of 18–39 (pre-presbyopic) and being capable of seeing 20/20 in both eyes separately with or without glasses correction. Subjects were excluded if they did not meet the inclusion criteria, if they had been diagnosed with any eye diseases or conditions like cataracts or keratoconus, and if they had previous refractive surgery such as LASIK or PRK. There were no exclusions for the spherical component of the refractive error, but the cylindrical component was restricted to no more than − 1.25D. Measurements were performed at Midwestern University’s Eye Institute. The Declaration of Helsinki was strictly followed in all procedures. Study protocol and written informed consent was approved (protocol #AZ 761) by the Institutional Review Board of the Office of Research and Sponsored Program from the Midwestern University at Glendale, AZ. Written informed consent was obtained from every participant.

Habitual refractions were recorded for all subjects. Habitual refraction is defined as the current prescription worn by the subject. Sixteen of the eighteen subjects presented with no more than − 1.00D of astigmatism correction in either eye. Participants responded to a subjective questionnaire (See Additional file 1) on their ocular history, the presence of any night vision problems while driving, and a rating of the severity of their symptoms on a scale of one (slightly bothersome) to ten (most bothersome).

A manifest subjective refraction was performed using a standard vision testing lane with mirror projection. The end point of refraction was minimum minus or maximum plus for maximum visual acuity followed by binocular balancing. A single investigator (licensed OD) performed subjective refraction for all the subjects. Visual acuity was tested using a logMAR chart from M&S Smart System Standard with black letters on a white background. Standard room lighting used in a typical eye exam was used on all subjects [6]. Measurements of pupil size in dim (~ 9 lx) and bright (~ 370 lx) illumination were also performed.

The ocular surface health was evaluated to ensure that there was no evidence of ocular surface disease (dry eye) and that the patient’s tear layer was healthy and free of debris. In addition, a simple macular photostress test was performed on each subject. During this test a bright light was projected into each eye. The duration taken for vision to recover to 20/20 was noted. The purpose of this test was to ensure that the study subjects were within normal limits for the test. Subjects were also pre-screened via questionnaire and a brief ocular heath examination was performed.

Measurements were obtained using the i.Profiler^plus^ for each subject. The i.Profiler^plus^ readings included measurements of low, high-order, and total aberrations at small (3 mm) and maximum pupil sizes at an average illuminance of 22 lx. The i.Scription software interfaced with the i.Profiler^plus^ and was used with the subjective refraction to compute a final i.Scription result [10, 11]. The examiner who did the subjective examination, did not perform the iProfiler^plus^ measurements or have access to the information. Since iProfiler^plus^ is an objective measurement calculated by the device, knowledge of the subjective measurement might not have an influence. When multiple measurements were taken from iProfiler^plus^, the first image was selected when all images were of similar quality with similar pupil sizes. If images were not similar in quality, only the best measurement was chosen such that the ring and sensor images showed minimal discontinuities (often due to tear film breakup or interference of the eyelids).

### Data analysis

All the data were manually captured on a recording sheet. This data was then entered into excel for some of the data analysis. SPSS (IBM) and BoxPlotR (online version) was used to calculate boxplots [18]. First, normality of the data was assessed using Shapiro Wilk test. Habitual, subjective and i.Scription refraction were compared using t-tests. Correlation between optical aberrations and refraction was performed. Scores from the questionnaire for the symptomatic vs asymptomatic subjects were compared statistically. Since i.Scription is derived from subjective refraction, most of our analysis involves comparing habitual prescriptions to the i.Scription. Statistical significance were set with a *p*-value of 0.05.

## Results

The means, standard errors, and standard deviations of the spherical, cylindrical, and sphero-cylindrical refractions were calculated and summarized in Table 1.

**Table 1:** Summary of the spherical, cylindrical and sphero-cylindrical i.Scription and habitual refractions

Refraction component	Mean	Std. Error	Std. Deviation
i.Scription Spherical	−1.58	0.62	2.62
i.Scription Cylindrical	−0.57	0.09	0.36
i.Scription Sphero-cylindrical	−1.86	0.60	2.55
Manifest Spherical	−1.65	0.62	2.64
Manifest Cylindrical	−0.42	0.09	0.37
Manifest Sphero-cylindrical	−1.86	0.60	2.56
Habitual Spherical	−1.75	0.62	2.62
Habitual Cylindrical	−0.27	0.08	0.35
Habitual Sphero-cylindrical	−1.88	0.60	2.55

**Fig. 1:**
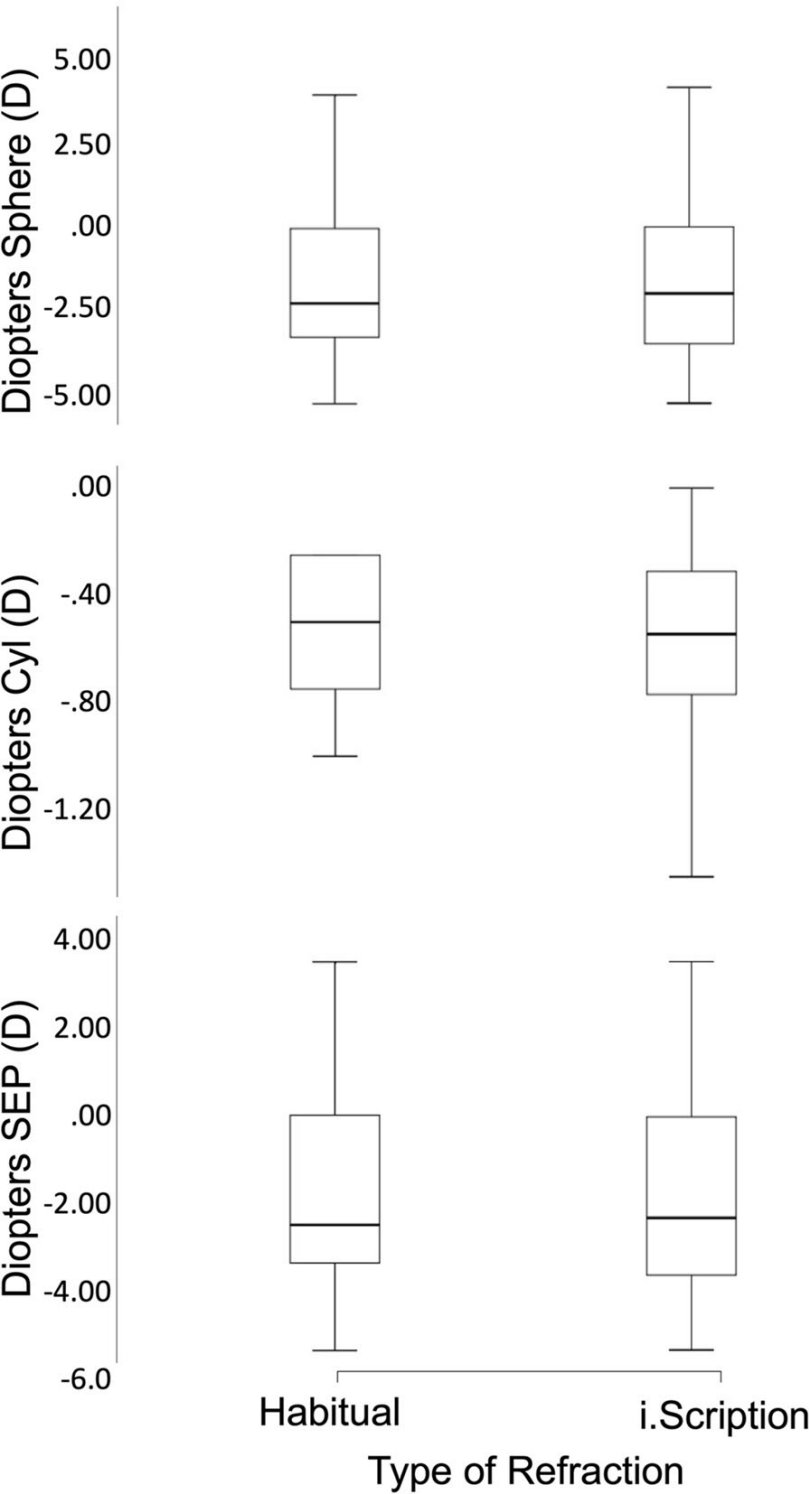
Plot of spherical refraction (**a**), cylindrical refraction (**b**), and spherical equivalent (SE) refraction (**c**) comparison among habitual and i.Scription. Center lines show the medians; box limits indicate the 25th and 75th percentiles as determined by SPSS software; whiskers extend 1.5 times the interquartile range from the 25th and 75th percentiles, outliers are represented by dots; data points are plotted as open circles. *n* = 18 sample points

**Fig. 2:**
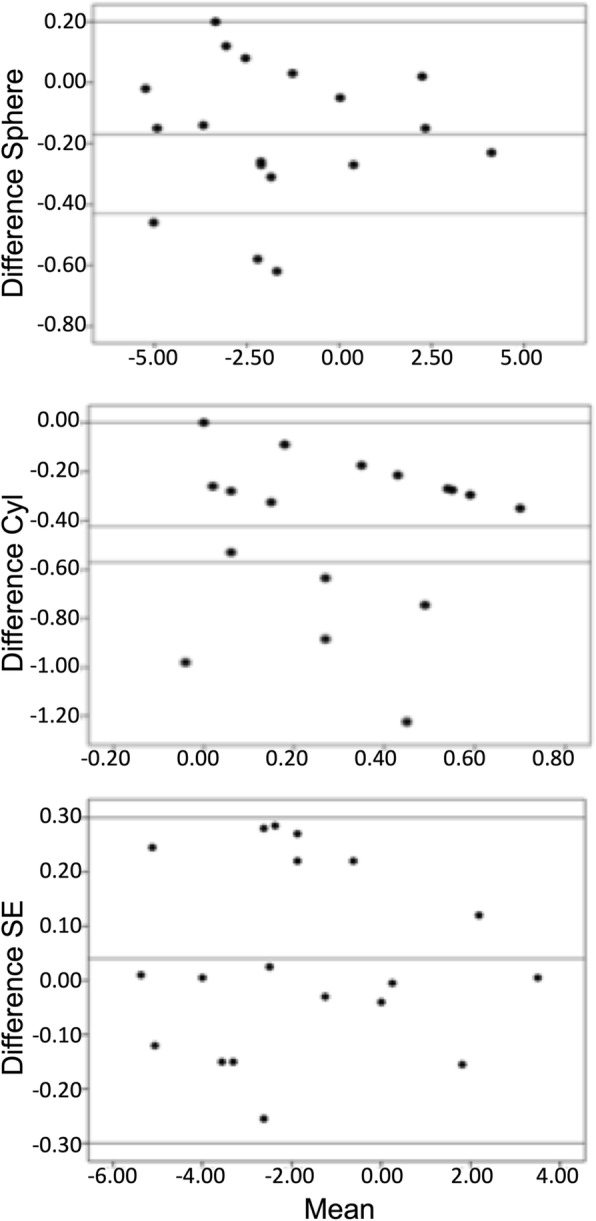
Bland-Altman plot comparing habitual and i.Scription refraction from the spherical refraction (**a**), cylindrical refraction (**b**), and spherical equivalent (SE) refraction (**c**). The differences are plotted in Y-axis and the average in the x-axis

Normality was assessed using Shapiro Wilk test before the parametric tests were run. Paired t-tests (Table 2) were performed to compare habitual and i.Scription, subjective and i.Scription, and habitual and subjective refractions for the spherical, cylindrical, and spherical equivalent (SE) components of the refractive error. For the spherical comparison, the habitual and i.Scription difference was significant (t = 3.13, *p* < 0.01) as was the subjective and i.Scription comparison (t = 2.32, *p* = 0.03), but the habitual and subjective difference was not significant (t = 1.80, *p* = 0.09). This was also true for the cylindrical comparison, the habitual and i.Scription difference was significant (t = − 5.40, *p* < 0.01) as was the subjective and i.Scription comparison (t = − 2.55, *p* = 0.02) but the habitual and subjective difference was not significant (t = 2.05, *p* = 0.056). There was no significant difference for the spherical equivalent with either the habitual and i.Scription comparison (t = 0.57, *p* = 0.58), the subjective and i.Scription comparison (t = − 0.68, *p* = 0.51), or the habitual and subjective comparison (t = 0.70, *p* = 0.50). Figure 1a-c illustrates these differences between habitual and i.Scription for the Spherical (a), Cylindrical (b), and SE (c) components. Table 2 summarizes these t-test comparisons between the habitual, subjective, and i.Scription refractions for the spherical, cylindrical, and spherical equivalent refractions. A Bland-Altman analysis was performed and is illustrated in Fig. 2a-c. The spread in the data is within the confidence interval.

**Table 2:** Paired t-tests to compare Habitual vs i.Scription and Subjective vs i.Scription refractions for sphere, cylinder, and spherical equivalent (SE)

	t Stat	*p*-value
Spherical t-test Comparison		
Habitual-i.Scription	3.12	< 0.01
Subjective-i.Scription	2.32	0.03
Habitual-Subjective	1.80	0.09
Cylindrical t-test Comparison		
Habitual-i.Scription	5.40	< 0.01
Subjective-i.Scription	2.55	0.02
Habitual-Subjective	2.05	0.06
SE t-test Comparison		
Habitual-i.Scription	0.57	0.58
Subjective-i.Scription	0.68	0.51
Habitual-Subjective	0.70	0.50

There was a strong correlation between the spherical equivalent (SE) i.Scription refraction and total RMS ocular aberration values for the 3 mm (*p* < 0.01, r = − 0.72) and maximum (*p* = 0.01, r = − 0.57) pupil sizes. The magnitude of the difference between the SE i.Scription and the SE habitual refraction was correlated to the high-order RMS for the 3 mm pupil size (*p* < 0.02, r = 0.48) and the maximum pupil size (*p* < 0.01, r = 0.64). Significant correlations were found between the spherical i.Scription and the total RMS for the 3 mm (*p* < 0.01, r = − 0.71) and maximum (*p* = 0.01, r = − 0.58) pupil sizes. Significant correlations were also found between the spherical equivalent (SE) i.Scription and total RMS for the 3 mm (*p* < 0.01, r = − 0.73) and maximum (*p* < 0.01, r = − 0.60) pupil sizes. No other significant meaningful correlations were observed.

**Fig. 3:**
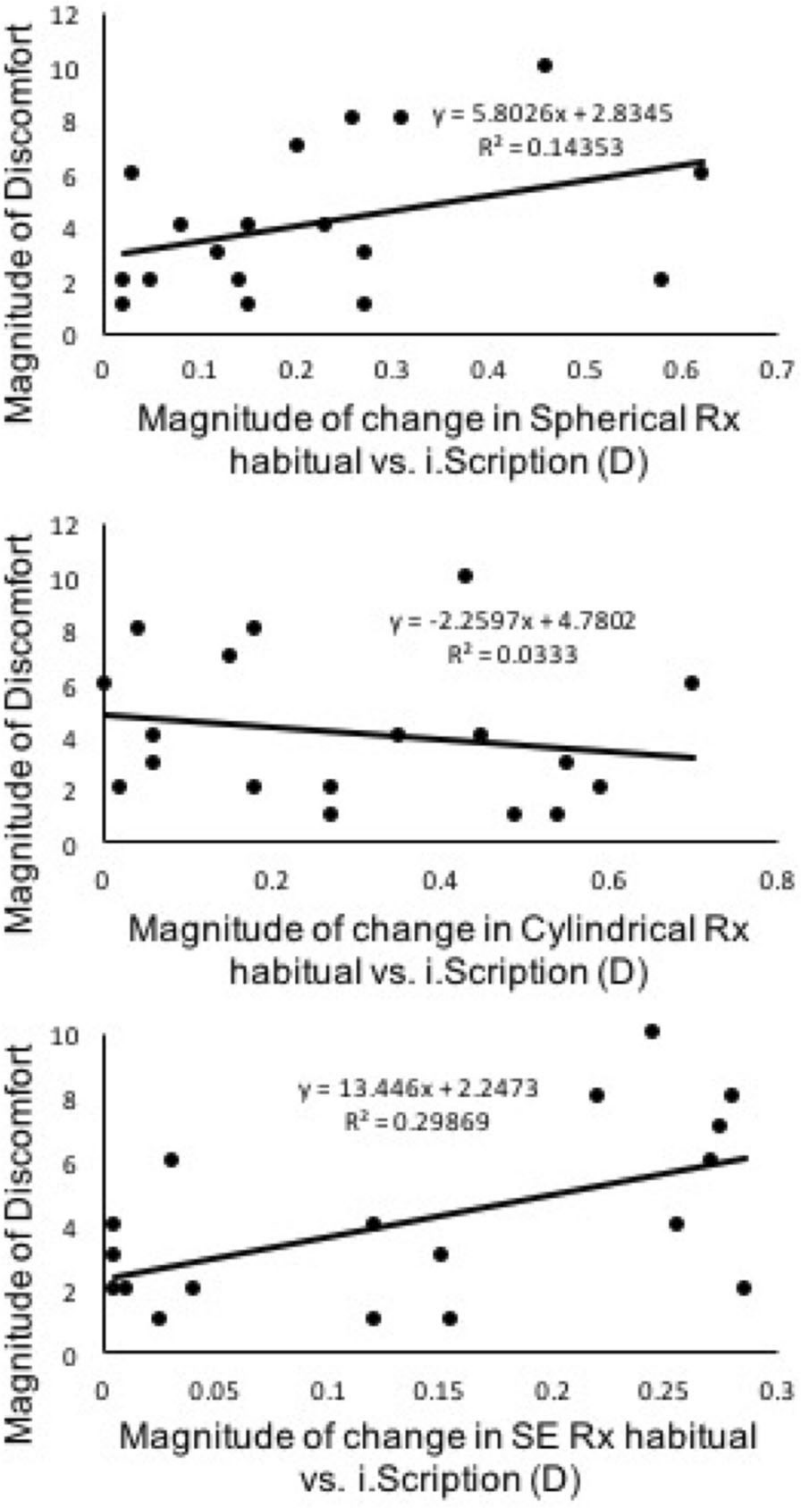
Plots comparing the magnitude of discomfort (on a scale of 1 to 10 where 10 was most bothersome) as a function of the magnitude of the difference in refraction between i.Scription and habitual corrections for the spherical refraction (top), cylindrical refraction (middle), and spherical equivalent (SE) refractions (bottom)

Subjective symptoms were identified from the questionnaire. Ten out of the eighteen subjects had discomfort at night. The mean (SD) of the magnitude of discomfort for all subjects was 4(2.7) on a numeric scale of one to ten with ten being the worst. Subjects who had difficulty with driving reported a mean difficulty score of 5.8 (*n* = 10) and the asymptomatic had a mean score of 1.8 (*n* = 9). Based on an independent t-test on this sample, they were significantly different (t = 4.3, *p* = 0.01). Regression analysis demonstrated a relationship between the magnitude of discomfort and the magnitude of the difference in SE i.Scription and SE habitual prescriptions (*p* = 0.02, r = 0.55). This is illustrated in Fig. 3. No difference was found between the magnitude of discomfort and the magnitude of the difference in spherical (*p* = 0.12, r = 0.38) or cylindrical prescriptions (*p* = 0.47, r = 0.18). This difference was also reflected in a correlation analysis, which was significant for the comparison of magnitude of discomfort vs. the difference in SE refractions between habitual and i.Scription (p = 0.01), but not for the spherical and cylindrical component differences. A sub-analysis of myopic subjects (15/18) revealed an increase in the significance of the correlation between the magnitude of discomfort vs. the difference in SE refraction (*p* = 0.002) while all other correlations were not significant. These results are summarized in Table 3. There was a correlation between the maximum pupil size and the magnitude of discomfort (*p* = 0.11, r = − 0.38).

**Table 3:** Correlations for the comparison of the subjective rating of discomfort to the difference in refractions between the Habitual vs. i.Scription refractions for the spherical, cylindrical, and spherical equivalent (SE) components

Refraction component	All Subjects Pearson Correlation (*n* = 18)	All Subjects *p*-value (*n* = 18)	Myopic Subjects Pearson Correlation (*n* = 13)	Myopic Subjects *p*-value (*n* = 15)
Difference spherical (habitual vs. i.Scription)	0.51	0.08	0.40	0.21
Difference cylindrical (habitual vs. i.Scription)	−0.42	0.13	−0.50	0.16
Difference SE (habitual vs. i.Scription)	0.75	0.01*	0.95	0.002*

*Correlation is significant at the 0.05 level

## Discussion

There were some interesting findings in the study and the initial findings were presented at the American Academy of Optometry’s Annual Meeting in 2017 [19]. Some statistically significant differences were found when comparing the spherical and cylindrical components of the habitual and i.Scription refractions and the subjective and i.Scription refractions. No statistically significant difference was found between the spherical equivalent (SE) powers. Since the i.Scription uses manifest refraction to determine the final prescription, as expected, they were not statistically different from each other (*p* = 0.51) for the spherical equivalent refraction. If a patient’s i.Scription prescription is significantly different from manifest refraction or their habitual prescription it may be one of the reasons for a patient to have visual complaints or other compliance issues.

Manifest refraction can correct for most of the low-order aberrations, leaving behind the high-order optical aberrations [20–22]. Normal eyes have on average a root-mean-square (RMS) error of 0.33 μm for high-order aberrations at a pupil size of 6 mm [23]. This is equivalent to 0.25D of defocus. Spherical aberration creates local refractive errors proportional to the square of radial distance from the pupil center. Pupil margin can be as much as 3D more myopic than the pupil center for an 8-mm pupil. Manifest refractions are dominated by the pupil center [24]. The goal of the i.Scription device is to generate a prescription that would be very accurate, in comparison to the manifest refraction as well as possibly eliminate any visual disturbances by compensating for high-order aberrations [25]. i.Scription sphere and cylinder powers are calculated to the nearest 0.01D step, thereby making it more precise than conventional prescriptions. A very recent study [26] investigated the impact of Seidel aberrations on optimum refractive state when discriminating small bright lights on a dark background. Using adaptive optics to correct and introduce optical aberrations, the investigators reported the presence of myopic shifts with positive spherical aberrations and hyperopia with negative spherical aberrations. An individual with a 7 mm pupil size and 0.16D/mm^2^ might experience as much as − 1.5D of shift in refraction at night while gazing stars [26]. These are the individuals that could benefit from i.Profiler type refraction and i.Scription based spectacles to do these specific tasks. Key to their treatment is producing a custom correction that reduces or eliminates the higher order aberrations under larger pupil size instead of adding a standard corrective shift to their habitual correction.

There was a strong correlation between the i.Scription refraction and the low-order RMS wavefront error for the 3 mm and maximum pupil sizes. Subjective refractions and conventional autorefractors typically correct to the nearest quarter of a diopter, most of which is represented by the low-order aberrations described by this low-order RMS wavefront error value [27]. Hence, it is a reassuring finding that the i.Scription refraction also correlates well with this measure. Similarly, there was a strong correlation between spherical equivalent i.Scription and total RMS for both 3 mm and max pupil size. In addition, there was a strong correlation between spherical component i.Scription and total RMS for both 3 mm and maximum pupil size. These findings are similar to that from Charman [28].

There was a correlation between the subjective rating of discomfort and the magnitude of the difference in the spherical equivalent refraction between the habitual and i.Scription refractions. This correlation was not significant for the spherical or cylindrical components on their own. In addition, a sub-analysis of myopic subjects showed an increase in the significance of this relationship. Larger differences in spherical equivalent refraction were correlated with more discomfort, suggesting that for our subjects and in particular our myopic subjects, the interaction between the spherical and cylindrical components may play a larger role than either component on its own in terms of the impact on the visual discomfort experienced by our subjects.

The real benefit using these lenses would arise when an individual finds it increasing difficult to perform their normal tasks using their habitual correction at lower light levels where the pupil is dilated to 6 mm or larger and optical aberrations play a larger role. These i.Scription derived spectacles can also be useful in individuals with larger magnitudes of optical aberrations during photopic conditions (e.g., keratoconus).

### Limitations

There are few limitations in the current study. While this study demonstrated no statistical difference in prescription between the i.Scription and the manifest refraction, future studies are warranted to determine if there would be a perceived improvement in vision when comparing spectacles made in a particular lens design with the i.Scription vs. free form digital lenses ground in the same lens design using the manifest refraction. In addition, there was a smaller sample size. Larger sample studies should be performed to understand this better.

## Conclusions

The i.Scription method improves night vision by correcting the sphere and cylinder more precisely. The i.Scription based refraction has the potential to improve the visual performance of certain tasks under either photopic or scotopic conditions. There was a correlation between the amount of change in the cylinder value between habitual and i.Scription prescriptions and the magnitude of the reported visual discomfort at night.

## Additional file

Additional file 1: Intake Form. (DOCX 14 kb)

## Abbreviations

RMS: Root Mean Square
SD: Standard Deviation
SE: Spherical Equivalent
