# Supplementary material for: Comparing habitual and i. Scription refractions
Source: BMC Ophthalmol. 2019 Feb 12;19:49. doi: 10.1186/s12886-019-1053-x (PMC6373049; doi:10.1186/s12886-019-1053-x)
Supplement: Supplementary file 1 — Intake Form. (DOCX 14 kb) [file 12886_2019_1053_MOESM1_ESM.docx]

**Intake Form**

**Please fill out the following questions as thoroughly as possible.**

1. When was your last eye exam? ___________________________________________
2. Do you wear glasses? _________________________
   1. Approximately how old is your current pair of glasses? _____________________
   2. Approximately how often do you wear your glasses? ______________________
   3. How is your vision through your glasses? If it’s poor, describe why. __________________________________________________________________________________________________________________________________
   4. Do you have any concerns with the fit or comfort of your current glasses? __________________________________________________________________________________________________________________________________
   5. Do you have an antireflective coating on your glasses? ____________________
3. Do you wear contact lenses? _____________________
   1. What brand of contact lenses do you wear? _____________________________
   2. Approximately how often do you throw your contacts away? ________________
   3. Approximately how many hours a day do you wear your contacts? ___________
   4. What contact solution(s) do you use? ___________________________________
4. Do you have trouble with night driving? ___________________________
   1. Describe what bothers you most about driving at night. ___________________________________________________________________________________________________________________________________________________________________________________________________

_________________________________________________________________

- 1. Do you find that you have more difficulty with driving in very dark conditions, at dusk/dawn, or in both conditions? ________________________________________________________________
  2. The majority of the time do you tend to wear your glasses, your contacts or neither when driving at night? ________________________________________________________________

1. Have you ever been diagnosed with dry eye? _________________________________
   1. Have you ever been diagnosed with ocular allergies? _____________________
   2. Circle any of the following symptoms that you experience on a weekly basis:

Dryness Tearing/watering Crusting of lids/lashes in the morning

Itching Burning Stinging Sandy/gritty feeling

- 1. Do you use drops in your eyes? _____________________________
  2. If yes to c, what is the name of the drops? _____________________________
  3. When is the last time you used the drops in your eyes? ___________________
  4. How long have you lived in Arizona? __________________________________

1. Have you ever been diagnosed with any eye diseases or conditions? ________________
   1. If yes, please specify: __________________________________________________________________________________________________________________________________
   2. Have you ever been told you have cataracts? ____________________________
   3. Have you ever been told you had keratoconus or other corneal ectasias?_______
2. Have you ever had any refractive eye surgeries such as LASIK or PRK? ______________
   1. Have you ever had any other eye surgeries? _____________________________
3. Are you taking any medications or herbal supplements? If yes, please list them below. Certain medications may cause blurry vision.

____________________________________________________________________________________________________________________________________________________________________________________________________________________________________________________________________________________________

Thank you for answering these questions as thoroughly as possible. This information will not be shared, and will be used strictly to analyze the results that we obtain in our study.
